# 

**Published:** 1820-05

**Authors:** 


*Lectures on Anatomy.*—J. C. Carpue, F.R.S. will commence his Lectures on Anatomy, Physiology, Pathology, and Surgery, at his Theatre, Dean-street, Soho, on Saturday, the 8th of June, 1820. The structure of the human body will be explained, as also its physiology and pathology. The operation of surgery will be shown on the dead body. Every day some part of the human body is demonstrated to the pupils, who are required to demonstrate in their turn. (This plan obliges Mr. C. to limit the number of his pupils.) Myology is not taught till the pupils are perfectly instructed in osteology, &c. A general examination takes place every tenth day; and, if the pupils do not perfectly recollect the parts that have been lectured on, they are again demonstrated.

The dissecting-room will be open from seven o'clock in the morning till five in the evening. The pupils are here taught the method of operating, as also the art of injecting and making preparations.

